# Supplementary figures and images for: Identification of a reliable fixative solution to preserve the complex architecture of bacterial biofilms for scanning electron microscopy evaluation
Source: PLoS One. 2020 May 29;15(5):e0233973. doi: 10.1371/journal.pone.0233973 (PMC7259777; doi:10.1371/journal.pone.0233973)

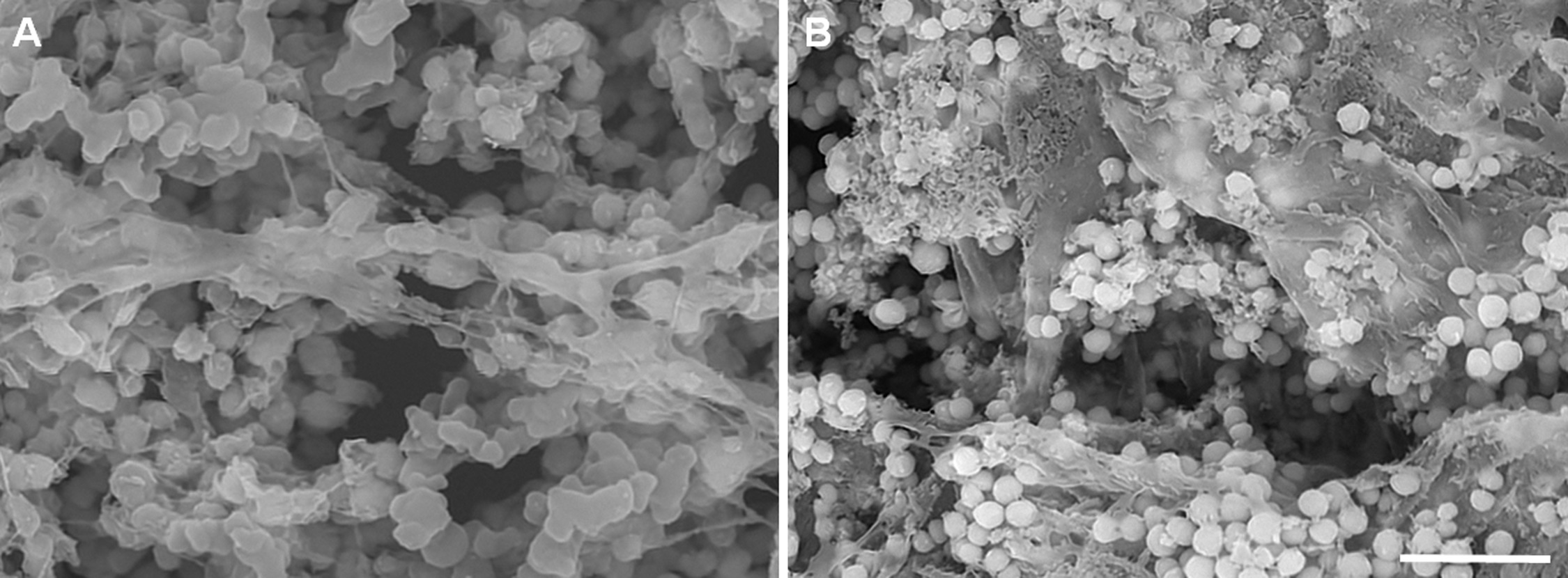

Supplement: S1 Fig — M. haemolytica (D153), grown in colorless RPMI 1640 (A) and S. aureus Newbould 305 (NB305), grown in BHI broth (B) on round glass coverslips in 24-well plates at 37°C for 48 h (NB305) to 72 h (D153). Biofilms grown on glass coverslips were fixed with Methacarn fixative solutions for 48 h and samples were further processed for SEM examination. One representative SEM biofilm image out of two independents experiments performed in triplicate wells are shown. (Bar = 4 μm; 12,000 magnification). (TIF) [file pone.0233973.s001.tif]
